# Evaluation of clinical characters and use of alternative medicines in the management of headache and predictors of treatment satisfaction among Saudi adults – A community-based study in Saudi Arabia

**DOI:** 10.1016/j.pmedr.2024.102787

**Published:** 2024-06-09

**Authors:** Alya Alghamdi, Mashael Eidhah Alsufyani, Falah Ali Alwadei, Hussam Abdullah Alshehri, Osama Samarkandi, Wajid Syed

**Affiliations:** a Community and Mental Health Nursing Department, King Saud University, Riyadh, Saudi Arabia; b King Saud University, Assistant Director of Administration at the College of Nursing, Riyadh City, Saudi Arabia; c Department of Basic Science, Prince Sultan College for Emergency Medical Services, King Saud University, Saudi Arabia; d Department of Clinical Pharmacy, College of Pharmacy, King Saud University, Riyadh, Saudi Arabia

**Keywords:** Headaches, Complementary medicine, Herbal medicine, Massage therapy, Cupping therapy, Nurses, Health care professionals

## Abstract

**Background and objective:**

The use of alternative medicine (AM) is rising globally. Hence this study aimed to assess the Use of and Satisfaction with Alternative Medicine in the management of headache and Related Factors in Individuals, living in Saudi Arabia.

**Methods:**

A cross-sectional study was conducted from July to December 2023 using an online questionnaire in Riyadh Saudi Arabia. The data was collected using a series, of prevalidated questionnaires aimed to assess the utilization and satisfaction of AM for the headache. Convenience sampling was applied for data collection.

**Results:**

A response rate of 93.1 % (n = 550) was obtained. Among the respondents, 78.7 % reported having headaches, while 69.2 % had a history of using AMs for headaches. However, among the users of AMs, 65.9 % of them were satisfied with the results of AMs, of those who were satisfied, 33.7 % said that AMs helped to prevent headache attacks, and 53.8 % reported that AMs use reduced pain intensity. The regression results demonstrated that there was a significant relationship between the satisfaction of AM for the headache and age (p < 0.004), and Active in sports (p < 0.019) and severity of the headache pain (p < 0.081)

**Conclusion:**

The findings reported that relatively high prevalence of AMs used to manage headaches, while 65.9% of the users were satisfied with the outcomes, the satisfaction was found to have a significant relationship with age, activity in sports and severity of pain. To avoid negative effects of AM, it is recommended to use under the qualified healthcare professionals.

## 1 Introduction

In the past few decades, alternative medicines have gained popularity and are being utilized globally to treat many conditions, including headache discomfort (Complementary and Health, 2018, Licina et al., 2023, Wei and Hsieh, 2023). According to the National Center for Complementary and Integrative Health (NCCIH), alternative medicine (AM) is a non-prescription practice used in conjunction with traditional medicine (Complementary and Health, 2018). On the other hand, integrative medicine is an approach to healthcare that acknowledges the advantages of incorporating complementary therapies (Yoga, massage therapies) that have been proven to be safe and effective with conventional procedures like medication and surgery (Aljawadi et al., 2020, Ng and Lau, 2020). According to literature complementary and integrative medicines are either natural products or mind–body practices (Complementary and Health, 2018, Phutrakool and Pongpirul, 2022). In this view, natural products include herbs, vitamins, and minerals, while mind–body practices include yoga, cupping therapy, chiropractic, massage, and acupuncture (Complementary and Health, 2018, Phutrakool and Pongpirul, 2022).

Herbal preparations include plant components, other ingredients, or mixtures are known as herbal medicines. (Al-Yousef et al., 2019, Barnes et al., 2004, Ng and Lau, 2020). The prevalence of AM for neurological diseases varies depending on the severity of the disease and the availability of the medicines (Al-Hashel et al., 2018, Polat and Sarilar, 2021, Wells et al., 2011). For instance, a previous study among US adults reported that 13.5 million in the United States with severe headaches reported utilizing at least one complementary AM treatment, which is 49.5 % more than the 33.9 % of adults without severe headaches (Wells et al., 2011). Similarly, another study among Turkish patients with headaches reported using phototherapy, cupping, and chiropractic adjustment as AM for the headaches (Polat and Sarilar, 2021). In Kuwait, a study reported that 69.9 % of the individuals who suffered from headaches used traditional medicine and Hijama, was the most common one (Al-Hashel et al., 2018).

Although the prevalence of headaches is rising globally with an estimated 40 % of the world's population, or 3.1 billion individuals, experiencing headaches in 2021, among those females are more likely than males to experience headache (Steinmetz et al., 2024). According to literature the prevalence of headache in Saudi Arabia was 65.8 % (Alshareef and Alsharif, 2023). The negative consequence associated with headaches was loss of interest in work or any activity, which resulted in absence of work and loss of job productivity in addition to disability which causes a burden on personal health (Alobaid et al., 2023, Magnavita, 2022). The treatment and management of the headache involve the use of conventional medicine or a combination of AM (Robbins, 2021). Since AMs pose minimal risks compared to conventional medicine, they are often prescribed alongside conventional medicine for headache management in the hope of controlling headache onset (Gaul et al., 2009). Frequently, patients seek alternative treatments to manage their diseases because they are unsatisfied with conventional medical treatments that they believe to be ineffective or harmful (Gaul et al., 2009, Syed et al., 2022).

Patient satisfaction with treatment can be defined as how satisfied the patient is with the treatment's ability to meet their health needs (Kromer et al., 2017, Schaarschmidt et al., 2018). Alternatively, it expresses the patient's feelings on specific elements of the treatment such as the duration of the therapy, as well as the therapeutic outcome (Kromer et al., 2017, Salame et al., 2018, Schaarschmidt et al., 2018). The literature suggested that satisfaction with the traditional AM was reported to be higher (Peltzer and Pengpid, 2018). For example, a study of 32 countries revealed a 71.9 % of the patients were satisfied with traditional and AM, and the satisfaction was between 71–72.7 % (Peltzer and Pengpid, 2018) various factors trigger the headache like inadequate diet, lack of physical activity, constant stress, hormone imbalance, and poor sleep, high workload, could all lead to headaches, that may be controlled, to some extent, using AMs (GöKSEL, 2013, Millstine et al., 2017, Syed et al., 2022). Furthermore, there is no study examining the satisfaction of AM use for headache management in Saudi Arabia. Therefore, this study aimed to assess the use of and Satisfaction with Alternative Medicine in the management of Neurological Disease and Related Factors in living in Saudi Arabia.

## 2 Methodology

### 2.1 Study design, setting and population

A web-based cross-sectional study was carried out in the Riyadh region of Saudi Arabia between July to December 2023 to evaluate the utilization and Satisfaction of AM for the management of neurological disease, and associated determinants among individuals. It was a self-reporting study that included adults (18 years of age or older), both sexes, being Saudi nationals, willing to fill out a questionnaire, having experienced a headache during the last week, and having used AMs in any way to treat the headaches. Others who do not match the inclusion criteria or Saudi adults living in other countries, headaches accompanied by pregnancy, and individuals with chronic diseases were excluded from the study. Furthermore, before carrying out the study informed consent was obtained from the respondents.

### 2.2 Sample size estimation

Similar to earlier research, the sample size for this study was determined. The sample size for this study was generated using https://www.raosoft.com/samplesize.html, yielding a sample of 377, with a 95 % Confidence level and a 5 % predetermined margin of error, with an unknown population. Further, we surveyed 612 people to prevent sampling bias.

### 2.3 Study instrument

The self-reporting online survey was developed purposefully by the research team. The survey was built later using Google Forms and the data collection tool was a structured questionnaire, which was developed after an extensive literature review of the previous studies (Al-Faris et al., 2008, Gaul et al., 2009, Millstine et al., 2017). Four components make up the questionnaire. Seven items in the first part evaluated the age, gender, and educational attainment of the participants as well as some clinical details including the history of headaches and the level of discomfort. The second set of questions asked about the history of using AMs as well as the frequency of headaches experienced in a given week (e.g., 1–4 times, between 5–9 times, 10–14 times, 15–20 times, and > 20 times). In the third section, there were questions about how satisfied you were using AM to treat headaches (e.g., did the AM improve your headache?). Each of these surveys had multiple choice and binary scale items, and a five-point Likert scale was used to gauge satisfaction. The satisfaction score was calculated by using satisfaction items(n = 2) where neutral was given a 1 score, satisfied and very satisfied was given a score of 1, and not satisfied and don't know were given a score of 0. The overall mean satisfaction score was computed and further divided into good satisfaction (who scored > 50 % of the total score) and poor satisfaction (who scored < 50 % of the total score).

With the assistance of a native Arabic speaker, the questionnaires were translated utilizing forward and backward translation techniques into the language of the region. Two stages went into the validation of the questionnaire design. First, the correctness, content, and administration duration of the questionnaire were assessed by a research team comprised of two senior professors with preparation expertise and one researcher to assess the research tool. Second, a pilot research was carried out with a sample of 20 participants chosen at random to get their feedback on making the questionnaire simpler. The final study did not incorporate the pilot study's findings. Additionally, reliability was assessed using the Cronbach alpha, and a result of 0.75 suggested the questionnaires helped conduct the study.

### 2.4 Data collection

**Fig. 1:**
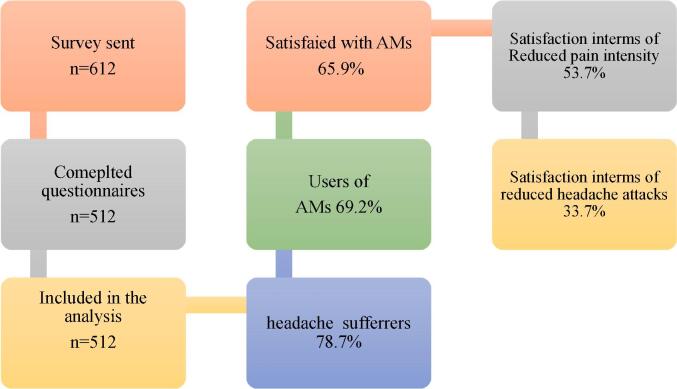
Responses among study respondents in Riyadh Saudi Arabia, from July to December 2023.

The final questionnaires were distributed online through the social media platforms such as WhatsApp, Twitter, and Facebook. Other methods to improve the response rate were considered such as reminders. The data collection followed convenience sampling methods, and it was restricted to the capital city of Saudi Arabia. Other methods such as snowballing were also used, where the survey was shared via friends and communities. The study enlisted the participation of 550 respondents. There were 512 valid responses were obtained in the study. Overall, included responses in this study were 512, giving a response rate of 93.1 %. The details of the descriptions of the responses are given in Fig. 1.

### 2.5 Data analysis

The statistical package for the Social Sciences (SPSS) version 27 for (SPSS Inc., Chicago, Illinois) was used. Descriptive statistics such as frequencies and percentages were computed. Univariate analyses (Chi-squared or Fisher exact test,) were used to test the difference between the variables. Furthermore, multiple linear regression was used to find out the predictors of satisfaction with alternative medicine. All statistical tests were performed at a significance level of α = 0.05.

## 3 Results

### 3.1 Description of the study population

A total of Five hundred twelve individuals provided a complete response, yielding an 83.7 % response rate (n = 612). The responses of females were higher than males 53.5 %(n = 274) vs 46.5 %(n = 238). In this study, 78.7 % of the respondents reported suffering from headaches, while 21.3 % of them did not have any headaches. When asked if they had experienced headaches in the previous week, 59.3 % of the female participants reported having them, compared to 40.7 % of the male participants. Additionally, 56.3 % of the participants in the 18–24 age group and 27.3 % of the participants in the 25–39 age group also reported having headaches. Table 1 summarizes the individual's comprehensive demographic responses on their headache state.

**Table 1:** Distribution of basic characteristics, according to headache status among Saudi adults in Saudi Arabia, from, July to December 2023, (n = 512).

**Variables**		**Do you experience severe headaches in the last week**	
		**Yes, n (%)**403 (78.7 %)	**No n (%)**109 (21.3 %)
Gender	Male	164(40.7)	74(67.9)
	Female	239(59.3)	35(32.1)
Age	18–24	227(56.3)	51(46.8)
	25–39	110(27.3)	29(26.6)
	40–54	57(14.1)	20(18.3)
	> 55	09(2.2)	09(8.3)
**Education**	Elementary	7(1.7)	08(7.3)
	Secondary	123(30.5)	05(4.6)
	University	256(63.5)	95(87.2)
	Postgraduate	17(4.2)	01(0.9)
**Psychological illness?**	Yes	59(14.6)	07(6.4)
	No	344(85.4)	102(93.6)
**Active in Sports**	Yes	176(43.7)	40(36.7)
	No	227(56.3)	69(63.3)

### 3.2 Frequency of headache, and pain rating

According to Table 2, 74.7 % of the respondents reported having headaches 1–4 times, whereas 13.4 % reported having headaches 5–9 times. In addition, 1.7 % of the headache sufferers said their pain was intolerable, and 43.7 % said their suffering was severe.

**Table 2:** Frequency of headache, and severity of the headache pain among respondents (n = 403).

***Variables***	***Frequency (n)***	***Percentage (%)***
**Frequency of headache**		
Between 1–4 times	301	74.7
Between 5–9 times	54	13.4
Between 10–14 times	22	5.5
Between 15–19 times	9	2.2
20 and above	17	4.2
**The severity of the headache pain**		
Unbearable pain	7	1.7
Very severe pain	38	9.4
Severe pain	176	43.7
Moderate	109	27
Mild	73	18.1

### 3.3 Frequency of headache, and association with gender and age

**Fig. 2:**
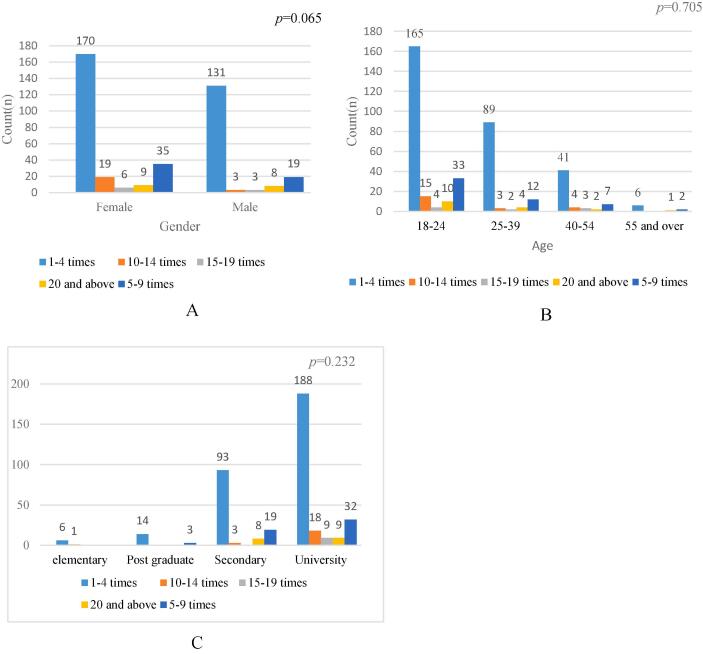
Frequency of headaches according to gender (Figure-2A), age (Figure-2B) and education (Figure-2c) among respondents, in Riyadh, Saudi Arabia in the past week from July to December 2023 (n = 403).

In this study, 170 females were reported 1–4 times of headache, compared to 131 of the males. Similarly, the frequency of 5–9 times was higher among females compared to males (Fig. 2A), however, there was no significant association was reported between the frequency of headache and age (p = 0.065). Similarly, the frequency of the headache was higher among individuals aged between 18–39 years compared to another age group, additionally, the frequency was higher among university individuals with a university degree, however, there was no significant association was reported between the frequency of headaches and age (Fig. 2B and education (Fig. 2C) (p = 0.05).

### 3.4 Severity headache, and association with gender, age, and education

**Fig. 3:**
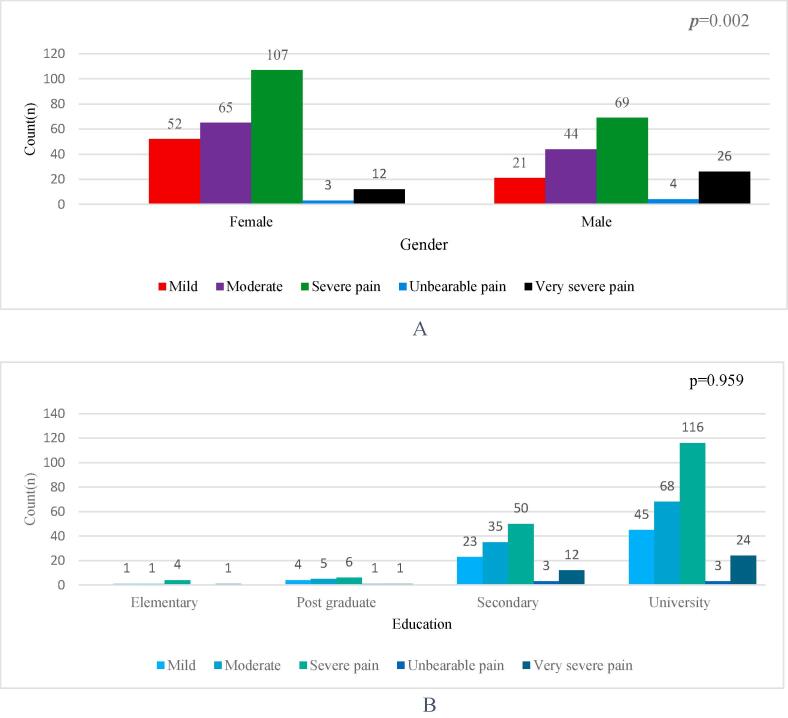
Severity of headache pain according to gender (Figure-3A) and education (Figure-3B) among respondents, in Riyadh, Saudi Arabia in the past week during July to December 2023 (n = 403).

The findings of this study show an association between the severity of the pain and gender (p = 0.002). For example, the severity of the headache pain was reported higher in females (n = 107) compared to males (n = 69), similarly the very severe pain was reported higher among males(n = 26) compared to females (n = 12) indicating a statistically significant association as shown in Fig. 3A.

Similarly, the severity of the headache pain was higher among individuals with university education, compared to individuals with other education, although there was no significant association was observed concerning the education age of the respondent’s severity of headache pain as shown in Fig. 3B.

### 3.5 Frequency, source, and side effects of AM use for the headaches

Among the respondents, 279 (69.2 %) had a history of using AMs for headaches, among the users of AMs, 189(67.7 %) used it based on advice received. Specifically, when asked respondents, who had been advised to use the AM for headaches,41.4 % of them said family and friends, while 10.9 % of the reported healthcare provider. Furthermore, 92.1 % of respondents reported having no side effects from utilizing AMs (Table 3).

**Table 3:** Frequency of responses towards the use of AM, sources of AM use, and response towards the incidence of side effects among respondents in Riyadh, Saudi Arabia, July to December 2023 (n = 403).

**Variables**	**Frequency**	**Percentage (%)**
	**(n)**	
**Have you ever taken an AM to prevent headaches?**		
Yes	279	69.2
No	124	30.8
**Did someone advise you to use AMs for your headaches (n = 279)?**		
Yes		
No	189	67.7
	90	32.3
**Who suggested you to use the AMs for headaches**		
Family or relatives	167	41.4
From the Internet or social networking sites	92	22.8
health care provider	44	10.9
Self-advice	100	24.8
**Have you noticed any adverse effects from using AMs?**		
Yes	22	7.9
No	257	92.1

### 3.6 Most commonly used alternative therapies for the headaches

The most commonly used alternative medicines among the respondents were relaxation techniques, in a quiet place or dark room 166 (22.5 %) followed by head and neck massage 177(24 %), use of caffeine, or herbal tea,149(20.1), cupping 121(16.4 %), and herbs 126(17.0 %) were the most commonly used AM therapies in this study.

### 3.7 Satisfaction with the use of AM

In this study, 65.9 % of respondents were satisfied with the results of AMs, of those who were satisfied, 33.7 % said that AMs helped prevent headache attacks, and 53.85 % reported that AMs use reduced pain intensity. According to Supplementary Table 1, 14.4 % of respondents were satisfied with the use of AM for headaches, and 3.0 % were completely satisfied with alternative medical treatment.

### 3.8 Satisfaction levels among respondents

In this study, 59 % of the respondents reported having good satisfaction with the use of alternative medicine, while 41 % of them reported poor satisfaction as shown in Supplementary Fig. 1.

To find out the relationship between the mean satisfaction of AM for the headache, and respondents' characters, a multiple linear regression model was utilized in which gender, age, activity in sports, and severity of the headache pain were considered as explanatory variables, and the mean score of satisfaction of AM for the headache was the dependent variable. There was a significant association between age, activity in sports, and severity of the headache pain with a mean score of satisfaction of AM for the headache. The results of the regression model demonstrated that there was a significant relationship between the mean score of satisfaction of AM for the headache and age (*p* < 0.004), and Active in sports (*p* < 0.019) and Severity of the headache pain (*p* < 0.081) as shown in Supplementary Table 2.

## 4 Discussion

In this study 78.7 % of the respondents reported having headaches, with regards to the intensity of the headache pain, 43.7 % of them reported severe pain, and 74.7 % of them reported a frequency of between 1–4 times a week. The severity of the headache pain was significantly associated with gender, where more females reported higher severity of the pain compared to males. These findings were similar to a previous study by (Al Ghadeer et al., 2021, Al Jumah et al., 2020, AlBarqi et al., 2022, Sabah et al., 2022), and Sabah et al, in other regions of Saudi Arabia. For example, AlBarqi et al reported an 88.5 % prevalence of headaches and concluded that headaches are common illnesses in Saudi Arabia (AlBarqi et al., 2022). Similarly, another study by Al Jumah et al reported a prevalence of 77.2 %, (Al Jumah et al., 2020) Sabah et al reported a 56.1 % prevalence of headaches (Sabah et al., 2022).

In this study, the AM use for headaches was 69.2 %, and, among the users of AMs, majority (92.1 %) of them did not report any adverse effects with the use of alternative medicine. While earlier studies reported similar findings (Al-Hashel et al., 2018, Lyu et al., 2024). For instance, a previous study in Kuwait reported that 69.9 % of the studied population used some form of traditional medicine for headache (Al-Hashel et al., 2018). According to a recent study among the Chinese population concluded that Chinese herbal medicine is beneficial for headaches when used continuously for 28 days or more (Lyu et al., 2024). Similarly, another National Health Interview Survey among United States adults (US) with headaches reported using alternative medicines more frequently for the management of headaches (Wells et al., 2011). These findings suggest that individuals seek alternative treatment approaches when traditional treatments fail to meet their needs, are too expensive, or have too many side effects.

In this study, respondents used relaxation techniques, in a quiet place or dark room followed by head and neck massage, use of caffeine, or herbal tea, cupping, and herbs as the alternative methods of treatment for their headache. While previous study in a neighboring country reported using Hijama as an alternative medicine (Al-Hashel et al., 2018). Similarly, another study by Robbins in 2021 revealed that headache treatment includes combination products of caffeine (Robbins, 2021). On the other hand, another study revealed that the use of cupping therapy for the management was various types of headaches was effective (Ersoy and Benli, 2020).In addition, literature revealed that the use of nutraceuticals and diet, acupuncture, cognitive behavioral therapy (CBT), biofeedback, and relaxation techniques all alternative approaches are useful and effective in the management of various headaches (Licina et al., 2023). Similarly, Aljawadi et al. found that the most popular alternative medicines among Arabs were herbal medicines, followed by acupuncture, honey, medical massage, manual manipulation of bones, and traditional bone setting (Aljawadi et al., 2020). Relaxation techniques work by controlling stress and pain responses, as well as reducing sympathetic arousal and muscle tension, which is the simplest of all alternative treatments (Licina et al., 2023). The reasons behind the use of these techniques may be due to the side effects or cost associated with conventional medicine or religious or cultural beliefs that were the cause of seeking alternative medicine. However, the most common justification for using massage therapy may have been the respondents ' favorable assessments of its advantages for reducing stress and alleviating discomfort. It was also extensively emphasized because of its practical techniques, which entail holding and moving body tissues and muscles while applying fixed or adjustable pressure. Massage therapy improves blood flow to the neck, shoulders, and back of the head while relieving stiffness in those regions. Additionally, massage treatment may aid in the relief of tension-related headaches (Gupta and Gaurkar, 2022).

In this study, 65.9 % of the respondents were satisfied with the AMs use for headaches, while these results were opposite to a similar study conducted among US adults reported that most of them were neutral with their current AM treatment (Lee et al., 2016). Similarly, another study by Bekkelund et al among headache suffers reported satisfaction with the AMs use and further reported improvement in headaches when using AMs (Bekkelund et al., 2014). Factors such as using the incorrect AM therapy not knowing how to use it, or using alternative approaches very short period could all affect how satisfied or unsatisfied is with AM. According to the present investigation, there is no correlation between the level of AM satisfaction for the headache and characteristics of respondents like gender, and severity of the headache rating. However, there was a significant association between the satisfaction level of AMs and use concerning the age group of the respondents. Similarly, the current findings show that the frequency of headaches was more among females, compared to males. Likewise, the severity of the headache pain was higher among females than males with a Significant difference between them. These findings were comparable to previous studies published in other countries. For instance, a previous study by, Bekkelund et al reported that 37 % of the patients benefited from AM, but AM use was not correlated with age, education, or use of medication (Bekkelund et al., 2014). Similarly according to Echiverri et al., aging is inversely related to headache days across the cognitive spectrum and is linked to a decrease in headache days when there is no complicating cognitive disease (Echiverri et al., 2018).

There are a few limitations to this study. First, the study was self-reported, cross-sectional, and conducted online, which could potentially compromise the reliability of our findings. Nonetheless, it may be presumed that headaches and the use of AM for headache therapy have been precisely tracked given the survey was anonymous and entirely voluntary. Second, the study's scope extended beyond hospital consultation patients to include the general public. Still, this study provides valuable background information on AM use among headache sufferers. Such a baseline is needed to determine the use pattern over time.

## 5 Conclusion

The findings reported that relatively high prevalence of alternative medicines used to manage headaches, while 65.9 % of the alternative medicines users were satisfied with the outcomes, the satisfaction of AM for the headache was found to have a significant relationship with age and activity in sports and severity of the headache pain. To avoid adverse reactions or negative effects associated with the use of alternative medicines it is recommended to use under the guidance of qualified healthcare professionals like doctors, nurses, or pharmacists. These findings also point to the need for additional research to determine why some headache patients seem to respond less effectively to present treatments, as well as the necessity of providing alternatives for those who are still unhappy with their available options. In addition, we advocate for the creation of educational programs that teach people how to use AMs in a way that minimizes side effects and maximizes therapeutic benefits.

## Funding

This study was supported by the Research Supporting Project, King Saud University, Riyadh, Saudi Arabia (RSPD2024R1099), which provided funding for this work.

## CRediT authorship contribution statement

**Alya Alghamdi:** Writing – review & editing, Writing – original draft, Methodology, Funding acquisition, Formal analysis, Data curation. **Mashael Eidhah Alsufyani:** Writing – review & editing, Writing – original draft, Methodology, Investigation, Funding acquisition, Formal analysis, Data curation. **Falah Ali Alwadei:** Writing – review & editing, Validation, Supervision, Software, Resources, Methodology, Funding acquisition, Formal analysis, Data curation. **Hussam Abdullah Alshehri:** Writing – review & editing, Validation, Supervision, Software, Resources, Project administration, Methodology, Investigation, Funding acquisition, Formal analysis, Data curation. **Osama Samarkandi:** Visualization, Validation, Supervision, Software, Resources, Project administration, Methodology, Investigation, Data curation. **Wajid Syed:** Writing – review & editing, Writing – original draft, Visualization, Validation, Supervision, Software, Resources, Project administration, Methodology, Investigation, Funding acquisition, Formal analysis, Data curation, Conceptualization.

## Declaration of competing interest

The authors declare that they have no known competing financial interests or personal relationships that could have appeared to influence the work reported in this paper.

## Data Availability

Data will be made available on request.
