# Supplementary material for: Evaluation of clinical characters and use of alternative medicines in the management of headache and predictors of treatment satisfaction among Saudi adults – A community-based study in Saudi Arabia
Source: Prev Med Rep. 2024 Jun 9;43:102787. doi: 10.1016/j.pmedr.2024.102787 (PMC11225024; doi:10.1016/j.pmedr.2024.102787)
Supplement: Supplementary Data 1 [file mmc1.docx]

**Supplementary Table** 1- Frequency of Participant's Satisfaction with the Use of AM for Headaches in Riyadh, Saudi Arabia from July to December 2023 (n=403)

| **Variables** | **Frequency**  **(n)** | **Percentage (%)** |
| --- | --- | --- |
| **Are you satisfied with the results of AMs (n=279)?**  Yes  No | 184  95 | 65.9  34.1 |
| **What is your opinion on the satisfaction with the AMs?**  I think somewhat satisfaction with the outcomes  Not satisfied  Satisfied  Very satisfied  I don’t know | 197  74  58  12  62 | 48.9  18.4  14.4  3.0  15.4 |
| **What is your opinion on the satisfaction with the use of herbal medicines?**  Satisfied  Not satisfied  I think somewhat satisfaction with the outcomes  Very satisfied  I don’t know | 64  61  223  55  0 | 15.9  15.1  55.3  13.6  0 |
| If yes, has AM helped you (n=184)  Reduces headache attacks  Reducing the pain intensity  I don’t know | 62  99  23 | 33.7  53.8  12.5 |

**Supplementary Figure-1 satisfaction levels of Alternative medicine for the headache**

**Supplementary Table 2 Regression results of mean satisfaction of AM for the headache, and respondents' characters**

| **Model** | **Unstandardized Coefficients** | | **Standardized Coefficients** | **t** | ***p-value*** | **95.0% CI for B** | |
| --- | --- | --- | --- | --- | --- | --- | --- |
|  | **B** | **Std. Error** |  |  |  | **Lower Bound** | **Upper Bound** |
| (Constant) | 1.659 | .172 |  | 9.669 | <.001 | 1.322 | 1.996 |
| Age: | -.086 | .030 | -.141 | -2.859 | .004 | -.144 | -.027 |
| Gender | -.071 | .050 | -.071 | -1.422 | .156 | -.170 | .027 |
| Active in sports | .112 | .047 | .116 | 2.355 | .019 | .018 | .205 |
| The severity of the headache pain | .045 | .026 | .087 | 1.748 | .081 | -.006 | .096 |

*CI= Confidence intervals
